# Supraglottic foreign body in a woman with Down's syndrome and congenital heart disease: A case report

**DOI:** 10.1097/MD.0000000000025455

**Published:** 2021-04-09

**Authors:** Yuchao Liu, Zijia Liu, Yang Zha, Xuerong Yu

**Affiliations:** a Department of Anesthesiology; b Department of Otolaryngology, Peking Union Medical College Hospital, Chinese Academy of Medical Sciences and Peking Union Medical College, Beijing, China.

**Keywords:** airway, anesthesia, congenital heart disease, Down's syndrome, foreign body

## Abstract

**Rationale:**

An impacted foreign body (FB) in the larynx of an adult is a rare but potentially life-threatening occurrence. Patients with Down's syndrome (DS) are vulnerable to airway FB. However, the anesthesia for FB removal can be challenging. This report describes a case in which a FB was impacted between the vestibular folds in an adult with DS, congenital heart disease, and a difficult airway.

**Patient concerns:**

A 41-year-old woman swallowed a piece of sharp-tipped wooden skewer presented with a sudden onset of aphonia, dysphagia, and an acute sore throat without respiratory difficulty. The patient had DS, congenital heart disease, pulmonary arterial hypertension, and severe obstructive sleep apnea–hypopnea syndrome. The airway evaluation indicated that ventilation and intubation would be difficult due to retrognathia, macroglossia, adenotonsillar hypertrophy, and Mallampati's classification III.

**Diagnosis:**

The clinical symptoms and laboratory examination confirmed FB penetrated between the vestibular folds.

**Interventions:**

After careful multidisciplinary preoperative assessment and preparation, the FB was removed successfully by direct laryngoscopy under moderate sedation and spontaneous ventilation, with the application of 1% lidocaine as topical anesthesia.

**Outcomes:**

The laryngeal FB was removed successfully without any complications. And the patient was discharged home the next day.

**Lessons:**

This case report shows the importance of anesthetic depth for laryngeal FB removal. The use of moderate sedation (allowing spontaneous ventilation) and adequate analgesia combined with local anesthesia enabled the patient to withstand the stress of direct laryngoscopy. Appropriate assessment, careful preparation, and multidisciplinary collaboration yielded the smooth removal of a laryngeal FB in an adult with DS.

## 1 Introduction

An airway foreign body (FB) is potentially life-threatening.^[1]^ FB aspiration is uncommon in adults, and adults with FB aspiration may have complex complications of psychoses and neurological disorders requiring management by a multidisciplinary team. In most cases, the aspirated FB settles in the tracheobronchial tree or is expectorated by coughing; thus, FB impaction in the larynx is rare, occurring only in ∼2% to 9% of adults with FB aspiration.^[2,3]^ Patients with DS are susceptible to airway FB due to mental retardation, macroglossia, pharyngeal muscle hypotonia, and swallowing disorders.^[4,5]^ Patients with DS commonly have airway disorders, including subglottic stenosis and upper airway obstruction caused by soft tissue crowding and smaller facial skeletal anatomy, which increases the difficulty of airway management and anesthesia.^[6]^ Herein, we report a rare case of a supraglottic fractured wooden skewer FB located between the vestibular folds in an adult with Down's syndrome (DS) and congenital heart disease (CHD). Multidisciplinary evaluation and intervention achieved successful FB extraction without intubation in a patient with DS, CHD, and a difficult airway.

## 2 Case presentation

**Figure 1:**
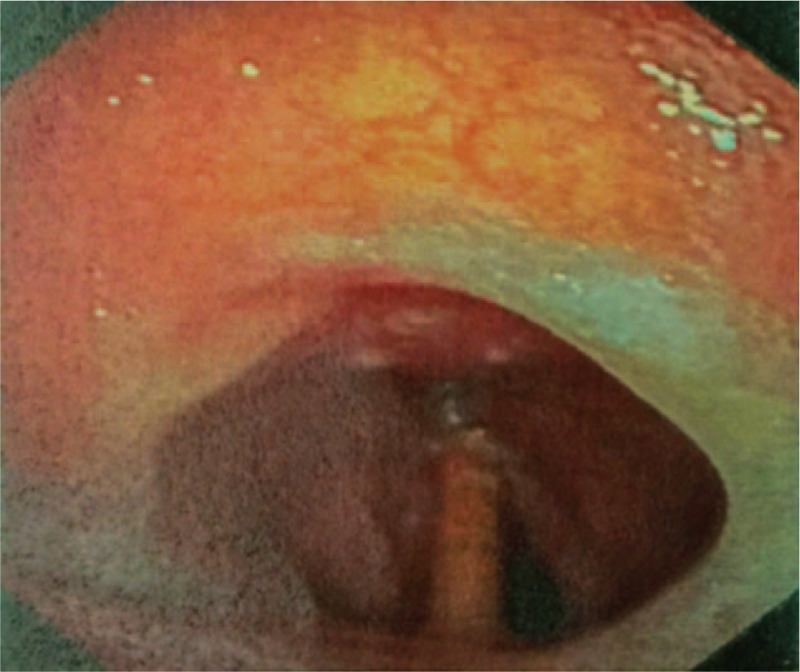
Endoscopic view of the larynx showing the wooden skewer firmly impacted between the two vestibular folds, with its anterior and posterior aspects embedded in the laryngeal mucosa.

A 41-year-old woman (height 150 cm, weight 50 kg) with DS and CHD accidentally ingested a fractured wooden skewer 20 h prior to presenting at the emergency department with aphonia, dysphagia, and an acute sore throat. A family member witnessed the sharp-tipped wooden skewer snap and then be swallowed by the patient. The patient seemed afraid and nervous, with no agitation. On physical examination, the patient had stable and normal vital signs without evidence of stridor and respiratory distress, but had a cardiac murmur and bilateral wheezing. Plain radiography showed no abnormalities except for mild patchy shadows in the bilateral lower lung fields. The patient tolerated a flexible laryngoscopic examination with the support of her family. Laryngoscopy provided visualization of a wooden skewer lodged anteroposteriorly between the two vestibular folds, with its end pointing anteriorly (Fig. 1). The position of the FB allowed air to be ventilated through the endolarynx and into the trachea easily. Echocardiography showed patent ductus arteriosus and moderate mitral valve insufficiency, with a left ventricular end-diastolic diameter of 63 mm and pulmonary arterial pressure of 58 mm Hg. The patient had a history of severe obstructive sleep apnea–hypopnea syndrome with retrognathia, macroglossia, adenotonsillar hypertrophy, and a normal active range of neck motion. The Mallampati's classification was III.

**Figure 2:**
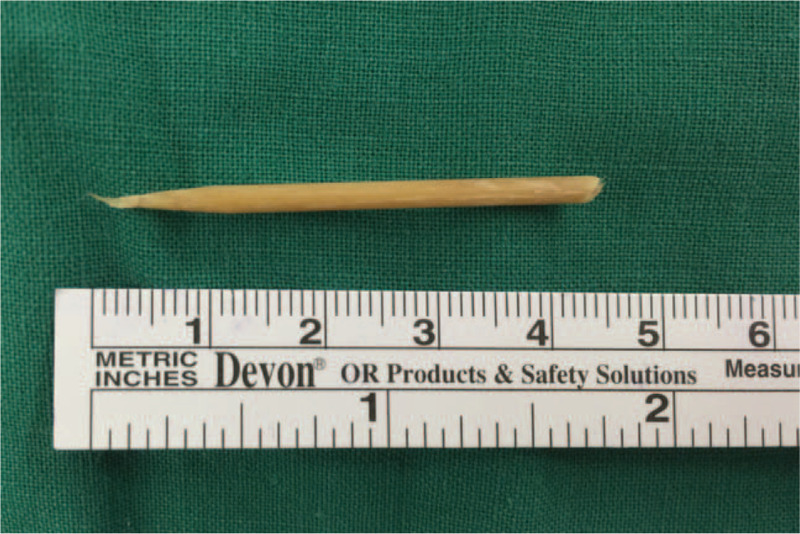
Photograph showing the wooden skewer after retrieval from the larynx.

The patient was admitted to the operating room without administration of any sedative or other pre-anesthetic medication. She was able to breathe without distress in the supine position. The anesthesiologist and nurses attempted to keep the patient calm while facilitating oxygen supply through a nasal cannula at 4 L/min and conducting venipuncture. Standard monitoring was performed, including oxyhemoglobin saturation, noninvasive blood pressure, and electrocardiography. Before the administration of anesthesia, the patient was premedicated with 5 mg of intravenous dexamethasone. A combination of dexmedetomidine and remifentanil was administered. Twenty minutes later, the patient was asleep but could be roused and could follow simple instructions, and was spontaneously breathing. This state of consciousness was maintained with dexmedetomidine 2 μg/kg/min and remifentanil 1 μg/kg/min. The anesthesiologist introduced a laryngotracheal topical anesthesia kit into the patient's mouth and sprayed topical 1% lidocaine on the surfaces of the oropharynx and the root of the tongue. After deepening the anesthesia with intravenous remifentanil (20 μg), the surgeon performed direct laryngoscopy, and successfully grabbed and removed the laryngeal FB with biopsy forceps. The FB was revealed to be a broken piece of wooden skewer with a length of 45 mm and a diameter of 2.5 mm (Fig. 2). A repeat flexible laryngoscopic examination showed no evidence of bleeding and only mild edema of the posterior commissure. The laryngoscopy procedure was completed in 6 min. Spontaneous ventilation was maintained throughout the procedure, without any decrease in oxyhemoglobin saturation. Before transferred back to ward, the patient was further monitored for 10 min until Steward recovery scores reached 6 and no respiratory support needed. The duration from induction to recovery was 40 min. The patient was discharged home <24 h later, without complications.

## 3 Discussion

A FB impacted in the larynx is a rare but critical condition that could quickly cause sudden complete airway obstruction requiring lifesaving first aid. ^[7]^ Fortunately, in the present case, the slender size of the skewer allowed the patient to maintain adequate oxygenation and ventilation. Furthermore, the definite witness helped with the timely and accurate diagnosis. Nevertheless, it was very challenging for the surgical team to remove the supraglottic FB in this patient with DS with poor coordination and a potentially difficult airway.

The treatment for laryngeal FB should be individually selected for each case. The method of anesthesia and surgical plan are dependent on the nature, size, and location of the FB, and the status of the patient. Aspirated FB are most commonly divided into organic materials (such as seeds and bones) and inorganic matter (such as dentures and metallic objects).^[8]^ Inhalation of a wooden FB is very uncommon, with such FB being prone to corrosion and breakage into small pieces. Although the slender shape of the FB in the present case seemed to avoid a life-threatening situation, the pointed end could have caused damage due to displacement or secondary edema. Therefore, the wooden FB had to be removed as soon as possible. The unusual location of the FB in the larynx made direct intubation impossible. However, the present patient was unable to cooperate with operative removal under local anesthesia, which is a favorable choice for adult patients in similar situations.^[5,9]^ Considering all these factors, the present case was carefully evaluated by a multidisciplinary team, including otolaryngology and anesthesiology, and the patient was scheduled to undergo emergency operative FB removal under sedation without a muscle relaxant with spontaneous respiration combined with local anesthesia. Open tracheostomy was planned as a back-up alternative in case the bleeding caused by the dislodgement of the FB worsened the respiratory status or the anesthesia was too deep and depressed spontaneous ventilation. Therefore, the surgical team was ready for immediate open tracheostomy, with the appropriate equipment prepared.

Maintaining an adequate anesthetic depth that did not depress the patient's respiratory drive was the key for the successful removal of the FB. The patient's history of obstructive sleep apnea–hypopnea syndrome and the airway assessment suggested that airway collapsibility or respiratory depression might result in ventilation difficulty, and even a disastrous airway emergency. However, the anesthesia needed to be sufficiently deep to minimize the patient's discomfort and prevent harmful reflexes, such as laryngospasm or dysphoria. Due to the limited patient cooperation, sudden coughing or bucking could have led to migration and deeper embedment of the FB, aggravating the damage and the difficulty of extraction. Furthermore, an excessive surgical stress response would have increased the risk of cardiovascular events in a patient with CHD. It was extremely challenging for the anesthesiologists to control the depth of sedation by titrating the anesthetics. The topical lidocaine also played an important role in suppressing the airway reflexes and reducing the required amount of general anesthetic,^[10]^ and the staff made persistent efforts to comfort the patient and minimize her irritation.

It was also essential to ensure that the patient was adequately oxygenated during the procedure. Auscultation and radiography indicated possible lung infections. Furthermore, pulmonary hypertension may be exacerbated by lack of oxygen, increasing the cardiac risk. Therefore, oxygenation was provided via a nasal catheter, and the oxygenation was closely monitored. A ventilation mask and open tracheostomy equipment were prepared to cope with a possible sudden airway emergency.

Multidisciplinary teamwork was vital to enable the FB to be removed without the need for a tracheostomy, and to prevent complications. In particular, it was extremely important to maintain good communication between the anesthesiologist and otolaryngologist both intra- and preoperatively. The carefully discussed anesthetic plan for the procedure comprised the spraying of topical 1% lidocaine, insertion of direct laryngoscopy, and other stimulation required to maintain sufficient anesthetic depth.

In conclusion, we presented a case in which an adult with DS, CHD, and a difficult airway had a supraglottic FB that was successfully removed under moderate sedation using a combination of dexmedetomidine and remifentanil, with spontaneous breathing maintained. This case report highlights the importance of controlling the appropriate anesthesia depth for supraglottic FB removal. For each individual airway FB, a detailed preoperative evaluation, sufficient preparation, and multidisciplinary teamwork are paramount to avoid serious complications.

## Acknowledgments

The authors would like to thank the patient's family for giving consent.

## Author contributions

**Conceptualization:** Yuchao Liu, Zijia Liu, Xuerong Yu.

**Investigation:** Yuchao Liu, Yang Zha.

**Resources:** Yang Zha.

**Supervision:** Zijia Liu, Xuerong Yu.

**Writing – original draft:** Yuchao Liu, Zijia Liu.

**Writing – review & editing:** Yuchao Liu, Zijia Liu, Yang Zha, Xuerong Yu.
